# The Relationship Between Functional Movement and Physical Health-Related Quality of Life in Active Adults

**DOI:** 10.3390/jfmk11040400

**Published:** 2026-10-02

**Authors:** David Kawahata, Duke Biber

**Affiliations:** 1 School of Natural & Applied Sciences, William Jessup University, Rocklin, CA 95765, USA; dkawahata@jessup.edu; 2 Department of Health Sciences, College of Health and Behavioral Studies, James Madison University, Harrisonburg, VA 22807, USA

**Keywords:** functional movement capacity, health-related quality of life, cardiorespiratory fitness, body composition, functional fitness

## Abstract

**Purpose:** To examine the relationship between functional movement capacity, cardiovascular capacity, and body composition, and how they relate to health-related quality of life. **Methods:** This was a cross-sectional observational study. In total, 123 adults completed the RAND Short Form-36 Health Survey, body composition, a 1.5-mile run, and four functional movement assessments. **Results:** Participants classified as functional or advanced function demonstrated significantly higher physical HRQoL than those classified as developing function or limited function across all movement patterns. **Discussion:** Functional movement capacity is an important indicator of perceived physical health and may provide a practical assessment tool for identifying individuals who may benefit from targeted exercise interventions.

## 1. Introduction

Functional movement capacity (FMC) refers to the ability to efficiently perform fundamental movement patterns that are essential for overall health and well-being including the effective execution of everyday tasks and activities [1]. Functional movement represents the integration of mobility, stability, strength, balance, coordination, and motor control into purposeful movement behaviors that support occupational, recreational, and daily living activities. Unlike isolated measures of fitness, functional movement reflects how effectively a person can use their physical capacities in real-world environments. Consequently, FMC has emerged as an important construct in exercise science because it captures the interaction among multiple physiological systems rather than assessing a single fitness component in isolation. As individuals age or become increasingly sedentary, impairments in movement quality may contribute to reductions in physical function, activity participation, and overall health status. Therefore, evaluating functional movement may provide valuable insight into an individual’s current level of physical functioning and potential future health risks [1].

The Functional Movement Screen (FMS) is a screening tool designed to evaluate fundamental movement patterns, mobility, stability, and asymmetries within the body through a series of seven standardized movements. It was developed to help identify dysfunctional movement patterns that may increase injury risk or limit physical performance. The primary purposes of the FMS are to guide corrective exercise strategies, improve movement efficiency, assist in injury prevention, and provide coaches and healthcare professionals with a systematic method for assessing functional movement capacity. The FMS has been shown to be a predictor of health-related quality of life in the general population [2]. Beyond injury prevention, movement screening procedures may help practitioners identify functional limitations that are not readily apparent through traditional measures such as body weight, body composition, or aerobic fitness testing. An individual may demonstrate adequate cardiorespiratory fitness yet still experience substantial limitations in mobility, postural control, or movement efficiency that affect daily functioning. Consequently, movement assessments are increasingly being incorporated into health promotion and wellness programs as a means of evaluating physical function from a more holistic perspective. Because movement quality influences participation in both structured exercise and routine daily activities, understanding functional movement capacity may provide important insight into overall health and well-being.

It is important to note that the present study did not administer the FMS. The FMS is described here because it represents the most established precedent linking movement competency to HRQoL and because it illustrates the general rationale for movement-based assessment; the four assessments used in the present study are distinct from the FMS in both content and scoring format, and findings from the FMS literature should not be assumed to transfer directly to them. The rationale for the specific assessments selected is described below.

A related construct to functional movement capacity is health-related quality of life (HRQoL), which is a multidimensional construct commonly used to characterize subjective well-being within the general population and serves as an important patient-reported outcome measure in healthcare research and practice [3]. Evaluation of HRQoL requires consideration of individuals’ perceptions, experiences, and the complex physical, psychological, and social dimensions that contribute to overall well-being [4]. Patient-reported outcomes may differ substantially from clinician-based assessments and objective health indicators, emphasizing the importance of incorporating patients’ perspectives when assessing health status and quality of life [5]. Health-related quality of life has become increasingly important in both clinical and public health research because traditional physiological indicators alone do not fully capture how individuals experience their health. Although objective tests can quantify disease risk, physical fitness, or functional limitations, they often fail to assess the day-to-day impact that these factors have on an individual’s ability to live a meaningful and satisfying life. HRQoL measures help bridge this gap by capturing subjective perceptions of physical functioning, emotional well-being, social participation, vitality, and overall health. As healthcare systems increasingly emphasize patient-centered outcomes, HRQoL has become a critical measure for evaluating intervention effectiveness and overall health status.

Recent research supports the relationship between HRQoL and various health outcomes, such as cardiorespiratory fitness [6] and body composition [7]. Individuals with higher levels of aerobic fitness generally report better physical functioning, greater vitality, and more positive perceptions of health when compared to less fit individuals. Similarly, favorable body composition has been associated with improved mobility, lower incidence of chronic disease, and enhanced physical functioning, all of which contribute to quality-of-life outcomes. These findings support the notion that physical health is multidimensional and that several physiological characteristics may collectively shape perceptions of well-being. Understanding the relative contributions of fitness, body composition, and movement competency may therefore provide a more comprehensive understanding of factors associated with HRQoL.

While HRQoL and FMC are correlated in the elderly [8] and in clinical populations [9], there is less consensus on what functional movements are most crucial to HRQoL. A recent systematic review found low quality of evidence in a review of 3614 articles utilizing 20 instruments aiming to relate HRQoL and FMC [10]. This lack of consensus may be partially attributable to the substantial heterogeneity across studies, including differences in measurement tools, study populations, and operational definitions of functional ability. Furthermore, many investigations have focused on older adults or individuals living with chronic disease, which limits the generalizability of findings to physically active adult populations. Determining which movement patterns are most strongly related to quality of life may allow practitioners to select more meaningful screening tools and develop more targeted interventions aimed at improving physical function and perceived health. Additional research is warranted to clarify how functional movement influences quality of life among community-dwelling adults who are actively engaged in exercise and wellness pursuits.

That said, studies with varying populations emphasize the importance of functional task exercise and isometric holds for functional ability and HRQoL [1,11]. Functional task exercises closely resemble activities commonly performed in daily life and frequently require coordinated engagement of multiple muscle groups, joints, and movement systems. Likewise, isometric holds challenge postural control, muscular endurance, and body awareness in ways that may be relevant to everyday physical functioning. Movements such as squatting, balancing on one leg, crawling, and hanging require simultaneous contributions from strength, mobility, stability, and coordination, thereby providing an integrated assessment of overall movement competency. These movement patterns may be particularly useful because they can be performed with minimal equipment and are easily administered in fitness, rehabilitation, and community health settings. Consequently, practical movement assessments may offer clinicians and fitness professionals an efficient means of evaluating aspects of physical function that are closely related to quality of life.

### 1.1. Selection of Movement Assessments

Established movement batteries were considered but judged poorly suited to the present sample and research question. The Short Physical Performance Battery and related clinical measures were developed to detect functional decline in frail or older populations and demonstrate ceiling effects in highly functioning community-dwelling adults aged 40 years and older, limiting their capacity to discriminate across the upper range of function represented in this sample [12,13]. The FMS requires proprietary equipment and rater training, scores individual items on a compressed ordinal scale that yields limited variability, has shown weak convergence with objective measures of the joint mobility it is presumed to reflect [14,15], and has not demonstrated sufficient association with injury to be recommended as a predictive screening tool [16]. Because this study aimed to characterize graded differences in movement capacity among adults already engaged in regular exercise, assessments producing continuous, time-based scores were preferred over ordinal ratings.

The four assessments were selected because each samples a distinct capacity with established links to health and physical function. The primal squat reflects floor-level mobility and postural control; the ability to move to and from the floor integrates strength, flexibility, balance, and body composition and has independently predicted all-cause mortality in middle-aged and older adults [17]. The single-leg Romanian deadlift hold challenges unilateral balance and posterior-chain control; failure of even a brief one-legged stance is independently associated with mortality and adds prognostic information beyond established clinical risk factors [18]. The bear crawl requires contralateral coordination and trunk stability, and quadrupedal movement training has been shown to improve movement quality, active range of motion, joint position sense, and cognitive flexibility in active adults [19,20,21]. The dead hang indexes grip and upper-body isometric endurance, which predicts all-cause and cardiovascular mortality more strongly than systolic blood pressure [22] and demonstrates a dose–response relationship with SF-36 physical component scores but not mental component scores [23], a dissociation directly relevant to the present hypotheses. All four require minimal equipment and no rater certification, consistent with the applied orientation of this investigation. These assessments have not previously been validated as a battery, and their use here is therefore exploratory.

### 1.2. Purpose and Hypothesis

The purpose of this study was to examine the relationship between functional movement capacity, cardiovascular capacity, and body composition, and how they relate to health-related quality of life (QOL) in a physically active adult sample. It was hypothesized that functional movement capacity would be related to health-related quality of life, cardiovascular fitness, and body composition in adult exercisers.

## 2. Methods

### 2.1. Procedures

Adults who were members at a fitness center in Northern California were recruited to participate in this study. To be eligible for the study, participants must have been members of the fitness center, 18 years of age or older, willing and able to engage in the functional movement assessments and pass the Physical Activity Readiness Questionnaire (British Columbia Ministry of Health, Vancouver, BC, Canada). Participants were recruited via electronic email and social media posts through contact lists provided by the fitness center. Participants completed informed consent electronically via a Google Form prior to participation. Following informed consent, participants completed the body fat measure, SF-36, and the functional movement assessments in a private room at the fitness center, followed by the 1.5-mile run, which was conducted outdoors. Data collection occurred in spring 2026.

### 2.2. Measures

Participants completed the demographic questionnaire and SF-36 electronically via Google Forms, and the functional movement, body fat, and 1.5-mile run measures face-to-face at the fitness center.

### 2.3. Demographics

Participants responded to questions about gender and age.

#### 2.3.1. Body Fat

Body fat percentage was estimated from skinfold measurements obtained with Lange skinfold calipers (Cambridge Scientific Industries, Cambridge, MD, USA) by a single experienced administrator with more than 15 years of experience in skinfold assessment. Six sites were measured—chest, abdomen, thigh, triceps, subscapular, and suprailiac—with each site measured twice and the two values averaged.

Body density was estimated using sex-specific generalized equations: the Jackson and Pollock (1978) equations for men [24] and the Jackson, Pollock, and Ward (1980) equations for women [25], each incorporating the quadratic form of the skinfold sum together with participant age. Body density was converted to body fat percentage using age- and sex-specific two-compartment conversion constants; constants appropriate to each participant’s age and sex were applied, including the constants specified for women aged 60 years and older. A third estimate was obtained directly from sex-specific four-site equations based on the sum of abdomen, thigh, triceps, and suprailiac skinfolds and age. The final body fat percentage for each participant was calculated as the mean of these three estimates. Complete equations are provided in Supplementary Table S1.

Skinfold methods have demonstrated good predictive ability for estimating body fat percentage relative to underwater weighing, particularly in women [12,26], and Lange calipers have been reported to have favorable design characteristics for measurement accuracy compared with other caliper brands [26].

#### 2.3.2. Short Form 36 (SF-36) Health Survey (RAND Version)

This questionnaire (RAND Corporation, Santa Monica, CA, USA) assesses health-related quality of life over the past four weeks across eight key health domains, including physical functioning, role limitations due to physical health, bodily pain, general health perceptions, vitality (energy/fatigue), social functioning, role limitations due to emotional problems, and mental health [4]. Responses are converted to standardized scores ranging from 0 to 100, with higher scores reflecting better perceived health-related quality of life. Two summary T scores were generated for analysis: physical component score (PCS) and mental component score (MCS), each with a mean of 50 (SD = 10); higher scores are better.

#### 2.3.3. Primal Squat

Participants were instructed to hold a deep squat position with maximum available knee and hip flexion and maintain heels on the ground. Foot width was suggested to be approximately hip-width apart and there were no requirements for spinal alignment. Participants were instructed to hold the primal squat for as long as possible for up to 3-min. The primal squat was used to assess bilateral lower-extremity movement capacity, including mobility, postural control, balance, and coordination. The deep squat component of the Functional Movement Screen demonstrates good inter-rater reliability (ICC = 0.78), although reliability is lower for individual movements than for composite scores [14,27]. However, associations between squat scores and objective measures of joint mobility are generally weak, suggesting limited convergent validity [15]. Thus, the primal squat is best interpreted as a measure of overall movement quality rather than a direct assessment of specific joint mobility. Although the primal squat is not the FMS deep squat, as it is held for time rather than scored ordinally on a single repetition with a dowel held overhead, evidence from the analogous FMS item provides the closest available reliability estimate.

#### 2.3.4. Single Leg Romanian Deadlift Hold (SLRDL)

Participants were instructed to hold a SLRDL position where the non-grounded leg and head made a straight line and the stance leg had between 70 and 90 degrees of hip flexion. Maximum hold time was 90-s per leg or until the participant could not maintain balance. The total times for the right and left leg were averaged to one time for data analysis. Reliability of related SLRDL assessments has been variable, with reported intra- and inter-rater reliability ranging from slight to substantial depending on the rater, limb, and testing session [28]. Recent systematic evidence similarly indicates that individual movement-quality assessments can demonstrate substantially greater measurement variability than composite scores [14]. Consequently, the SLRDL hold appears appropriate for assessing unilateral functional movement capacity, but results should be interpreted cautiously given limited evidence of validity in general adult populations.

#### 2.3.5. Bear Crawl

Participants were instructed to bear crawl forward for 30-s and bear crawl backwards for 30-s three times without stopping or letting a knee touch the ground until exhaustion or 3-min. Participants were instructed to keep their upper torso level with the ground and step with the contralateral arm and leg. Direct evidence regarding the reliability and validity of observational bear-crawl movement-quality scores in adults is limited. Objective measures of bear-crawl characteristics have demonstrated good-to-excellent agreement with motion-capture measures, although agreement for some joint range-of-motion variables was weaker [29]. Therefore, the bear crawl should be considered an exploratory functional movement assessment, particularly when using investigator-developed scoring criteria.

#### 2.3.6. Dead Hang

Participants were instructed to hang on a straight bar with arms and legs straight with an overhand closed grip for as long as possible up to 2-min. Dead-hang assessments have demonstrated variable but generally favorable test–retest reliability, with reliability influenced by factors such as grip position, protocol, and termination criteria [30]. Evidence supporting construct validity is primarily derived from climbing populations, limiting generalizability to adults without climbing experience. Nevertheless, the dead hang provides a practical and objective measure of upper-body functional capacity when testing procedures are standardized.

#### 2.3.7. A 1.5-Mile Run

Cardiorespiratory fitness was assessed using a 1.5-mile timed run completed outdoors on a flat, marked 0.3-mile loop course; participants completed five laps to cover the full distance. The run was administered last in the testing sequence, after the body composition, questionnaire, and functional movement assessments had been completed. Participants were instructed to cover the distance as quickly as possible. Warm-up was permitted but not standardized; participants warmed up at their own discretion and no prescribed protocol was provided. Run times were recorded using handheld stopwatches operated by strength and conditioning coaches employed by the fitness center and by the first author. Depending on scheduling, participants began the run either individually or in small groups, with each participant’s time recorded by the administrator who started them.

All participants held current medical clearance and had completed a Physical Activity Readiness Questionnaire (PAR-Q) as part of the fitness center’s standard membership procedures. No participant was excluded from testing, no test was terminated prematurely, and all 123 participants completed the run. All testing was conducted on a single day under sunny conditions with ambient temperatures ranging from approximately 60 to 80 °F (16 to 27 °C). Participants completed their assessments at varying times between morning and evening, so time of day and ambient temperature differed across individuals, though the course and general weather conditions remained constant throughout.

#### 2.3.8. Functional Movement Composite Score (FMC4)

A composite Functional Movement Capacity score (FMC4) was created to provide a global gauge of overall movement competency across the four movement assessments. Each movement was classified into one of four ordered categories (1 = Advanced Function, 2 = Functional, 3 = Developing Function, and 4 = Limited Function) based on performance thresholds established by the first author prior to data collection (Table 1). These thresholds were derived from pilot testing conducted at a physical therapy clinic affiliated with and operating out of the participating fitness center, together with accumulated observational experience from the first author, the affiliated clinic, and the fitness center. No published criterion standards for these specific assessments were identified.

The thresholds were fixed before data collection and were not modified at any point thereafter. Following testing, each participant was provided with an individualized summary of their performance times and corresponding movement classifications; this feedback was distributed before any health-related quality of life data were examined, establishing that the classification scheme was fully specified independent of the outcome variable.

Each assessment was terminated at a predetermined maximum duration to maintain testing efficiency across the sample (primal squat and bear crawl, 180 s; dead hang, 120 s; single-leg Romanian deadlift hold, 90 s per leg). Participants capable of sustaining longer holds were stopped at the maximum. Consequently, the Advanced Function category for the primal squat and bear crawl corresponds to attainment of the protocol ceiling rather than to an independently derived criterion, and performance in the upper range of these distributions is right-censored.

Scores from the four movements were summed to create the FMC4 composite, with lower scores reflecting better overall movement capacity and higher scores reflecting greater movement limitation. The classification was created primarily for applied purposes: the participating facility provides individualized performance feedback to members, and ordered categories communicate movement status in a form that practitioners and participants can act upon, which a raw time in seconds does not. Summing the four classifications additionally reduced the number of separate predictors entered into the analysis. These are practical rather than psychometric justifications: the approach weights each movement equally and treats the four ordered categories as equally spaced, and neither assumption has been empirically established. The FMC4 is therefore presented as a practitioner-facing heuristic summary of movement competency rather than as a validated instrument, and analyses involving it should be interpreted as exploratory. Sensitivity analyses using continuous, threshold-free composites are reported in Section 3.4.

### 2.4. Data Analysis

An a priori power analysis was conducted using G*Power 3.1 (Heinrich Heine University Düsseldorf, Düsseldorf, DEU, UK) for the primary analysis examining whether functional movement capacity was related to physical health-related quality of life. The associations between functional movement capacity and physical health-related quality of life constituted the primary hypotheses and were specified prior to analysis. Additional subgroup comparisons and correlation analyses were exploratory and should be interpreted as hypothesis-generating. For detecting a medium-sized correlation (*r* = 0.30) with α = 0.05 and power = 0.80, the required sample size was 84 participants. This was a cross-sectional observational study. SPSS version 31 was used to analyze all data. Prior to conducting statistical analyses, data were screened for completeness, accuracy, and potential outliers. Descriptive statistics, including means and standard deviations, were calculated for all demographic, functional movement, fitness, body composition, and health-related quality of life variables. Pearson’s r correlation coefficient was used to determine relationships between all variables of interest. Correlation coefficients were interpreted to evaluate the strength and direction of associations among functional movement capacity, cardiorespiratory fitness, body composition, and health-related quality of life measures. An independent-samples *t*-test was conducted to compare physical health-related quality of life between the Functional/Advanced Function Group (Functional Group) and the Developing Function/Limited Function Group (Limited Function Group) for all four movements. An additional independent-samples *t*-test was conducted to compare mental health-related quality of life between the Functional Group and the Limited Function Group for each movement pattern. Group means, standard deviations, and test statistics were evaluated to determine differences in health-related quality of life between functional movement classifications.

A hierarchical (sequential) multiple linear regression was conducted to examine whether functional movement capacity, as measured by the FMC4, was independently associated with physical health-related quality of life (PCS). To control for the influence of established covariates, age (years), sex (1 = female, 2 = male), body mass index (BMI), and 1.5-mile run time (minutes) were entered in the first block of the model. FMC4 was entered in the second block to determine whether it explained additional variance in PCS beyond that accounted for by the covariates alone. Model fit was evaluated using R^2^ and adjusted R^2^ for each block, and the change in R^2^ (∆R^2^) associated with the addition of FMC4 in Block 2 was tested for statistical significance using the F-change statistic. Unstandardized (B and standardized (β) regression coefficients, along with corresponding 95% confidence intervals and *p*-values, were examined for each variable in the final adjusted model to determine the direction, magnitude, and statistical significance of the association between functional movement capacity and PCS, independent of age, sex, BMI, and 1.5-mile run time. Statistical significance for all analyses was established a priori at *p* < 0.05.

## 3. Results

### 3.1. Participants

A total of 123 adults participated in this study, with 79.7% being female (*N* = 98) and 20.3% being male (*N* = 25). The average age of participants was 43.2 years (*SD* = 10.22) and the average weight was 174.8 pounds (*SD* = 39.06). The average fat mass was 53.24 pounds (*SD* = 23.21) and body mass index was 28.1 (*SD* = 5.08). The average 1.5-mile run time was 15.50 min (*SD* = 4.34).

### 3.2. Correlations Between HRQoL and Functional Movement, Body Fat, and Fitness

There were statistically significant correlations between all variables and physical health-related quality of life (Table 2; *N* = 123, *M* = 50.2, *SD* = 5.99). The only variable that had a statistically significant correlation (*r* = 0.200, *p* = 0.027) with mental health-related quality of life (*N* = 123, *M* = 48.1, *SD* = 8.13) was the dead hang exercise; no other variable was significantly correlated with mental health-related quality of life. There were no statistically significant differences between male and female participants for either the physical component summary (*t* = 0.401(121), *p* = 0.689) or mental component summary (*t* = 0.058(121), *p* = 0.954).

### 3.3. Comparing HRQoL by Functional Movements

There was a significant difference in physical health-related quality of life between participants that had functional or advanced function ratings of functional movement capacity and those that had a developing or limited rating of functional movement capacity for all four movement patterns (Table 3). Mental health-related quality of life was higher in the Functional Group when compared to the Limited Function Group, but the difference was not statistically significant (Table 4). This differs from the correlational analysis reported in Section 3.2, in which dead hang time was significantly associated with mental HRQoL when analyzed as a continuous variable; dichotomizing performance into functional categories reduced statistical power to detect this association. Effect sizes for the mental HRQoL comparisons were small to moderate with confidence intervals spanning zero, indicating imprecise estimates rather than established absence of effect.

### 3.4. Comparing HRQoL by Overall Functional Movement Capacity

A composite score combining all four functional movements (FMC4) was calculated by summing the rating of each functional movement (see Table 1). The FMC4 (*N* = 123, *M* = 7.0, *SD* = 2.73) was significantly correlated with physical health-related quality of life (*r* = −0.452, *p* < 0.001). The regression model was statistically significant (*R*^2^ = 0.204, *F*(1, 121) = 31.045, *p* < 0.001). FMC4 significantly contributed to physical health-related quality of life (B = −0.990, 95% CI [−1.342, −0.638], β = −0.452, *t*(121) = −5.572, *p* < 0.001).

After controlling for age, sex, BMI, and 1.5-mile run time, functional movement capacity remained a significant independent variable of physical health-related quality of life (B = −0.670, β = −0.306, t(117) = −2.78, *p* = 0.006), accounting for an additional 5.0% of variance in PCS (∆F(1, 117) = 7.75, *p* = 0.006). This confirms functional movement capacity is independently associated with physical HRQoL. BMI was the only covariate to remain significantly associated with physical HRQoL (B = −0.28, β = −0.24, *p* = 0.037), while none of the other covariates included in the model were significantly associated with physical HRQoL in the final adjusted model, including age (B = −0.01, β = −0.02, *p* = 0.821), sex (B = −0.75, β = −0.05, *p* = 0.576), and 1.5-mile run time (B = −0.03, β = −0.02, *p* = 0.877).

To evaluate whether the observed association depended on the FMC4 classification thresholds, a sensitivity analysis was conducted in which the four raw performance times were standardized, summed, and re-standardized to form a continuous movement composite. The continuous and categorical composites were nearly perfectly correlated (*r* = −0.966, *p* < 0.001), with the negative sign reflecting the reversed scoring direction of the two measures. In a hierarchical regression using the same covariates, age, sex, BMI, and 1.5-mile run time accounted for 19.6% of variance in PCS (F(4, 118) = 7.191, *p* < 0.001). Adding the continuous composite in Block 2 explained an additional 4.9% of variance (∆F(1, 117) = 7.580, *p* = 0.007; total R^2^ = 0.245, adjusted R^2^ = 0.213). The composite was independently associated with physical HRQoL (B = 1.803, 95% CI [0.51, 3.10], β = 0.301, t(117) = 2.753, *p* = 0.007), such that each standard deviation increase in movement capacity corresponded to approximately 1.8 points higher PCS. BMI remained the only significant covariate (B = −0.294, 95% CI [−0.55, −0.04], β = −0.250, *p* = 0.024). These estimates were nearly identical to those obtained using FMC4 (β = −0.306, ∆R^2^ = 0.050), indicating that the association between functional movement capacity and physical HRQoL does not depend on the specific classification thresholds applied. Categorizing continuous predictors in regression reduces power and can introduce residual confounding [32,33]; the continuous model is therefore reported as the more conservative estimate, with the categorical model retained for applied interpretability.

To examine whether equal weighting within the composite obscured differential contributions across movements, the four standardized movement times were also entered as separate predictors in Block 2. This model explained an identical proportion of additional variance (∆R^2^ = 0.049), but the block did not reach significance (∆F(4, 114) = 1.851, *p* = 0.124) and no individual movement was independently associated with PCS (all *p* > 0.30; VIF 1.19–2.14). The four standardized coefficients were nearly identical in magnitude (β = 0.091, 0.108, 0.096, and 0.110 for the primal squat, dead hang, bear crawl, and single-leg Romanian deadlift hold, respectively), indicating that the equal weighting applied in the composite closely approximates the weighting produced by the data. These findings indicate that the association with physical HRQoL is carried by variance shared across the four assessments rather than by any single movement, and that aggregation confers a statistical advantage by concentrating shared variance into a single degree of freedom. Internal consistency across the four standardized assessments was acceptable (Cronbach’s α = 0.741; corrected item-total correlations 0.387 to 0.633), consistent with a set of related but deliberately distinct movement capacities. The primal squat showed the weakest association with the remaining assessments (item-total *r* = 0.387; α = 0.761 if deleted) and was retained because it provides the only assessment of bilateral lower-extremity mobility in the battery.

## 4. Discussion

The present study examined the relationships among functional movement capacity, cardiorespiratory fitness, body composition, and HRQoL in active adults. Overall, the findings demonstrated consistent associations between HRQoL and each of the functional movement assessments, body composition, and cardiorespiratory fitness. Participants classified as functional or advanced function reported higher physical HRQoL than those classified as developing function or limited function across all four movement patterns. Furthermore, the composite functional movement score significantly contributed to physical HRQoL, suggesting that movement competency represents an important related factor of perceived physical health. These findings are consistent with previous studies reporting positive associations between functional movement and HRQoL in community and clinical populations [2,8,9]. Likewise, the observed relationships between lower body fat, lower body mass index, faster 1.5-mile run times, and higher physical HRQoL align with previous evidence supporting the contribution of healthy body composition and cardiorespiratory fitness to overall well-being [6,7]. In contrast, functional movement capacity demonstrated relatively weak relationships with mental HRQoL, with only dead hang performance showing a statistically significant association. Although participants with higher functional movement ratings consistently reported higher mental HRQoL scores, these differences were not statistically significant. This finding suggests that physical function may be more closely aligned with the physical dimensions of HRQoL than with the psychological and social domains assessed by the SF-36. Cardiorespiratory fitness showed a significant unadjusted association with physical HRQoL (*r* = −0.307, *p* < 0.001), consistent with prior evidence linking aerobic fitness to perceived physical health [6]. In the adjusted model, however, 1.5-mile run time was not independently associated with PCS once age, sex, BMI, and functional movement capacity were accounted for. This pattern should not be interpreted as indicating that cardiorespiratory fitness is unrelated to physical HRQoL. Rather, it suggests that the variance in physical HRQoL shared with run time was also captured by the other variables in the model, particularly BMI and functional movement capacity, which are themselves correlated with aerobic fitness. Functional movement capacity, by contrast, retained an independent association with physical HRQoL after adjustment, indicating that it contributed information not accounted for by the covariates. Whether movement capacity is more closely related to perceived physical function than aerobic capacity, or whether the present measure of cardiorespiratory fitness was simply less precise, cannot be determined from these data.

### 4.1. Practical Applications

From an applied perspective, the findings suggest that simple functional movement assessments may provide useful information regarding physical function when incorporated into comprehensive health and fitness evaluations. The movement assessments examined in the present study require minimal equipment, can be administered efficiently, and reflect movement tasks commonly encountered in daily life. As such, assessments such as the primal squat, dead hang, bear crawl, and single-leg Romanian deadlift hold may offer practitioners a practical means of observing movement competency alongside more traditional measures of health and fitness. The observed associations between movement performance, physical fitness characteristics, and perceived health support the notion that functional movement assessments may contribute information that is not fully captured by measures of body composition or cardiorespiratory fitness alone. While traditional fitness assessments remain important indicators of physiological health, movement-based assessments may provide complementary information regarding an individual’s capacity to perform fundamental movement tasks involving mobility, stability, balance, strength, and coordination.

The findings also suggest that movement competency may represent a useful descriptive outcome measure within exercise, rehabilitation, and wellness settings. Monitoring changes in movement performance over time may provide practitioners with an additional perspective on physical function and responses to physical activity or training participation. However, the cross-sectional and exploratory nature of the present study precludes conclusions regarding causality, prognostic value, or the ability of these assessments to identify individuals who may experience future health, fitness, or functional outcomes. Furthermore, the findings do not validate these assessments as screening tools and do not establish clinical or performance thresholds for categorizing individuals, identifying elevated risk, diagnosing impairments, or directing exercise prescription. Rather, movement assessment results should be interpreted within the context of a broader evaluation that includes an individual’s health history, goals, physical fitness characteristics, and other relevant clinical or performance information. Future longitudinal and intervention-based research is needed to determine whether these functional movement assessments demonstrate meaningful utility for monitoring changes in physical function and whether they are associated with longer-term health, fitness, or quality-of-life outcomes. Until such evidence is available, these assessments should be viewed as complementary observational tools that may enhance, but not replace, established approaches to health, fitness, and functional evaluation.

### 4.2. Limitations

Several limitations should be considered when interpreting these findings. First, the cross-sectional design prevents any determination of causality, and it remains unclear whether greater functional movement capacity contributes to improved health-related quality of life or whether individuals with better perceived health are more likely to maintain higher levels of movement competency and physical activity. Second, participants were recruited through convenience sampling from a single fitness center in Northern California, creating the potential for selection bias and limiting the representativeness of the sample. The predominance of female participants (79.7%) further restricts the ability to generalize findings across sexes and limited our ability to examine potential sex-specific relationships. Third, although body composition and cardiorespiratory fitness were evaluated, the analyses did not account for several potentially important confounding variables, including physical activity volume, exercise history, injury status, chronic disease burden, pain, medication use, socioeconomic factors, sleep quality, and psychological characteristics, leaving the possibility of residual confounding. Measurement limitations should also be acknowledged. In addition, the functional movement classifications used in this study were based on predefined performance thresholds that have practical utility but have not been extensively validated as predictors of future health outcomes, injury risk, or quality of life. The FMC4 as a composite score lacks psychometrics and needs to be assessed for validity and reliability. Finally, because participants were relatively active adults already engaged in a fitness environment, the findings may not generalize to sedentary individuals, older adults, clinical populations, competitive athletes, or more diverse demographic and geographic populations. Therefore, the results should be viewed as preliminary and warrant confirmation through larger, multi-site, longitudinal studies employing more representative samples. The functional movement classification thresholds used in this study were derived from clinical observation and pilot testing rather than from validated criterion standards, and have not been evaluated against future health outcomes, injury risk, or quality of life in independent samples. Although the thresholds were specified a priori and sensitivity analyses indicated that the primary association did not depend on them, the specific category boundaries should not be adopted as diagnostic or prescriptive cut points, and their generalizability to other populations is unknown. Relatedly, the imposition of maximum test durations censored performance in the upper range for the primal squat and bear crawl, limiting discrimination among the most capable participants; this censoring would be expected to attenuate rather than inflate the observed associations. Several aspects of the cardiorespiratory fitness assessment also warrant caution. Run times were recorded by multiple administrators using handheld stopwatches, and inter-rater reliability for this measure was not assessed. Depending on scheduling, participants began the run either individually or in small groups; running in the presence of others may influence pacing and effort, introducing variability unrelated to cardiorespiratory capacity. Warm-up was not standardized, and although all testing occurred on a single day on the same course, participants completed the run at different times of day under ambient temperatures ranging from approximately 60 to 80 °F. The 1.5-mile run was also administered last in a testing sequence that included sustained isometric holds and a crawling assessment performed to volitional failure, and accumulated fatigue may have slowed run times relative to a rested performance. Because the testing order was identical for all participants, this would not be expected to confound between-participant comparisons, but these sources of variability may have attenuated the observed association between cardiorespiratory fitness and physical HRQoL. Finally, the comparisons of mental health-related quality of life were imprecisely estimated, with confidence intervals for several effect sizes spanning zero; these results should be interpreted as inconclusive rather than as demonstrating an absence of association.

### 4.3. Future Research

Future research should examine these relationships longitudinally to determine whether improvements in functional movement capacity result in corresponding improvements in HRQoL and to identify the mechanisms that may explain these associations [1,10]. Future studies should also investigate whether targeted interventions designed to improve movement competency lead to meaningful improvements in perceived physical health, physical activity participation, and functional independence. Additionally, examining these relationships across diverse populations, including older adults, sedentary individuals, athletes, and clinical populations, may provide a more comprehensive understanding of how functional movement influences quality of life throughout the lifespan. Further work exploring which movement patterns are most strongly associated with specific dimensions of HRQoL may help refine assessment strategies and inform the development of more effective exercise and health promotion programs.

### 4.4. Conclusions

In this sample of active adults, functional movement capacity demonstrated an independent association with physical health-related quality of life after adjustment for traditional indicators including body mass index, age, sex, and aerobic fitness. These findings support the inclusion of movement-based assessments as part of a more comprehensive evaluation of physical health and functioning. Additional longitudinal and intervention research is needed to determine whether enhancing functional movement capacity leads to meaningful improvements in health-related quality of life.

## Figures and Tables

**Table 1:** Functional movement ratings.

Movement	Limited Function		Developing Function		Functional		Advanced Function	
	Male	Female	Male	Female	Male	Female	Male	Female
Primal Squat (s)	<15	<15	15–45	15–45	46–179	46–179	≥180	≥180
Dead Hang (s)	<20	<15	20–45	15–30	46–89	31–59	≥90	≥60
Bear Crawl (s)	<30	<30	30–90	30–90	91–179	91–179	≥180	≥180
SLRDL Hold (s)	<15	<15	15–30	15–30	31–59	31–59	≥60	≥60

**Table 2:** Correlations between functional movements, body fat percentage, body mass index, and fitness with physical health-related quality of life.

Variable	*M*	*SD*	*r*	95% CI	*p*
Primal Squat (s)	143.0	64.54	0.238	[0.064, 0.398]	0.008 *
Dead Hang (s)	50.1	32.34	0.366	[0.203, 0.508]	<0.001 **
Bear Crawl (s)	131.5	54.39	0.383	[0.222, 0.523]	<0.001 **
SL RDL Hold (s)	68.5	23.53	0.332	[0.166, 0.479]	<0.001 **
1.5-Mile Run (min)	15.50	4.336	−0.307	[−0.457, −0.138]	<0.001 **
Body Fat Percentage	29.70	0.0789	−0.333	[−0.480, −0.167]	<0.001 **
Body Mass Index	28.1	5.08	−0.426	[−0.559, −0.270]	<0.001 **

Note. * *p* < 0.05; ** *p* < 0.001.

**Table 3:** Comparing physical health-related quality of life by functional movement categories.

Movement	Group	*n*	*M*	*SD*	*t* (*df*)	*p*	Mean Diff [95% CI]	Hedges’ g [95% CI]
Primal Squat (s)	Functional or Advanced Function	106	50.8	5.48	2.286 (18.72)	0.034	4.41 [0.37, 8.46]	0.75 [0.24, 1.27]
	Developing or Limited Function	17	46.4	7.65				
Dead Hang (s)	Functional or Advanced Function	82	51.5	4.81	3.152 (58.32)	0.003	3.93 [1.43, 6.42]	0.68 [0.30, 1.06]
	Developing or Limited Function	41	47.6	7.21				
Bear Crawl (s)	Functional or Advanced Function	89	51.4	4.77	3.236 (43.51)	0.002	4.49 [1.69, 7.29]	0.79 [0.38, 1.19]
	Developing or Limited Function	34	47.0	7.53				
SL RDL Hold (s)	Functional or Advanced Function	113	50.9	5.35	4.051 (10.00)	0.002	8.99 [4.05, 13.94]	1.63 [0.95, 2.30]
	Developing or Limited Function	10	41.9	6.84				

Note. Welch’s *t*-test reported for all comparisons given unequal group sizes and heterogeneity of variance; Levene’s test was significant for the primal squat, dead hang, and bear crawl comparisons [31]. Hedges’ g calculated using the pooled standard deviation.

**Table 4:** Comparing mental health-related quality of life by functional movement categories.

Movement	Group	*n*	*M*	*SD*	*t* (*df*)	*p*	Mean Diff [95% CI]	Hedges’ g [95% CI]
Primal Squat (s)	Functional or Advanced Function	106	48.3	7.83	0.332 (19.22)	0.743	0.85 [−4.50, 6.20]	0.10 [−0.41, 0.61]
	Developing or Limited Function	17	47.4	10.07				
Dead Hang (s)	Functional or Advanced Function	82	49.0	7.49	1.554 (67.58)	0.125	2.56 [−0.73, 5.86]	0.32 [−0.06, 0.69]
	Developing or Limited Function	41	46.4	9.14				
Bear Crawl (s)	Functional or Advanced Function	89	48.6	7.56	0.993 (49.96)	0.326	1.80 [−1.84, 5.43]	0.22 [−0.17, 0.61]
	Developing or Limited Function	34	46.8	9.46				
SL RDL Hold (s)	Functional or Advanced Function	113	48.5	7.76	1.401 (9.81)	0.192	4.98 [−2.96, 12.18]	0.62 [−0.03, 1.26]
	Developing or Limited Function	10	43.6	11.00				

Note. Welch’s *t*-test reported for all comparisons given unequal group sizes and heterogeneity of variance; Levene’s test was significant for the SL RDL comparison [31]. Hedges’ g calculated using the pooled standard deviation.

## Data Availability

Data is available on request from the authors.

## Supplementary Materials

The following supporting information can be downloaded at https://www.mdpi.com/article/10.3390/jfmk11040400/s1: Table S1: Equations used to estimate body fat percentage from skinfold measurements.
