# Supplementary material for: The Relationship Between Functional Movement and Physical Health-Related Quality of Life in Active Adults
Source: J Funct Morphol Kinesiol. 2026 Oct 2;11(4):400. doi: 10.3390/jfmk11040400 (PMC13692414; doi:10.3390/jfmk11040400)
Supplement: Supplementary file 1 [file jfmk-11-00400-s001.zip › jfmk-4532145-supplementary.pdf]

Supplementary Table S1

Equations used to estimate body fat percentage from skinfold measurements

Six skinfold sites were measured (chest, abdomen, thigh, triceps, subscapular, suprailiac), each twice, with the two values averaged. Three estimates of body fat percentage were computed for each participant and the mean of the three was used in all analyses.

Step 1. Body density (sex-specific)

Sex	Estimate	Equation
Men	BD1	BD = 1.10938 − 0.0008267(Σ_3) + 0.0000016(Σ_3)² − 0.0002574(age)Σ_3 = chest + abdomen + thigh
Men	BD2	BD = 1.1125025 − 0.0013125(Σ_3) + 0.0000055(Σ_3)² − 0.000244(age)Σ_3 = chest + triceps + subscapular
Women	BD1	BD = 1.0994921 − 0.0009929(Σ_3) + 0.0000023(Σ_3)² − 0.0001392(age)Σ_3 = triceps + suprailiac + thigh
Women	BD2	BD = 1.089733 − 0.0009245(Σ_3) + 0.0000025(Σ_3)² − 0.0000979(age)Σ_3 = abdomen + triceps + suprailiac

Male equations derived from Jackson and Pollock (1978); female equations derived from Jackson, Pollock, and Ward (1980). Skinfold values in millimetres; age in years.

Step 2. Conversion of body density to body fat percentage

Age- and sex-specific two-compartment conversion constants were applied. Estimates 1 and 2 were obtained by applying the appropriate constants to BD1 and BD2 respectively.

Group	Equation
Men, 20–80 years	%BF = (495 / BD) − 450
Women, 20–59 years	%BF = (496 / BD) − 451
Women, 60–90 years	%BF = (502 / BD) − 457

Step 3. Direct four-site estimate

A third estimate was obtained directly, without an intervening body density step, using the sum of four skinfolds (Σ_4 = abdomen + thigh + triceps + suprailiac).

Sex	Equation
Men	%BF = 0.29288(Σ_4) − 0.0005(Σ_4)² + 0.15845(age) − 5.76377
Women	%BF = 0.29669(Σ_4) − 0.00043(Σ_4)² + 0.02963(age) + 1.4072

Step 4. Final value

Reported body fat percentage = mean of Estimate 1 (from BD1), Estimate 2 (from BD2), and Estimate 3 (direct four-site).
